# PriBeL-Net: Extending betel leaf dataset with CNN-based image classification

**DOI:** 10.1016/j.mex.2026.103828

**Published:** 2026-02-15

**Authors:** Gauri Mane, Raghav Bhise, Rutuja Kadam, Gagandeep Kaur, Gitanjali Shinde, Grishma Bobhate, Sonal Fatangare

**Affiliations:** a Department of Computer Science Engineering – Artificial Intelligence and Machine Learning, Vishwakarma Institute of Information Technology, Pune, India; b Department of Computer Engineering, MIT, Academy of Engineering, Alandi, Pune, India; c Symbiosis Institute of Technology, Nagpur Campus, Symbiosis International (Deemed University), Pune, India; d Department of Computer Science Engineering – Artificial Intelligence and Machine Learning, Vishwakarma Institute of Technology, Pune, India

**Keywords:** Controlled Environment, On-Field, CNN models, Deep learning, Image classification

## Abstract

Deep learning is core to precision agriculture. In this research, the authors compare four deep-learning frameworks: MobileNetV2, EfficientNetB0, ResNet50V2, and DenseNet121, on a custom dataset under controlled and on-field environments.

Whereas MobileNetV2 and ResNet50V2 gave the best results on the controlled setup, robust to light variations, backgrounds, and different leaf orientations, DenseNet121 gave better results in field environments with good accuracy and F1-score.

However, EfficientNetB0 was not up to the mark, implying the restrictions of light-weight models while working with noisy, real-world datasets.

With such implications, DenseNet121 is reported to be the most dependable candidate for application in agriculture.

In the next phase, adaptation and rearrangement of DenseNet121’s architecture and parameters will be taken up to better its performance and its ability for adaptation under diverse agricultural conditions.

## Graphical abstract

**Figure:**
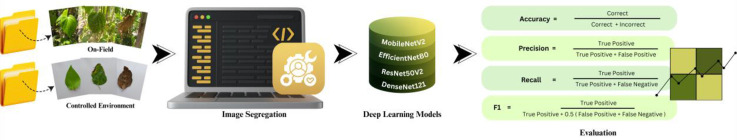

## Specifications table

**Table:** 

Subject area	Computer Science
More specific subject area	Betel Leaf Image Classification, Deep Learning
Name of your method	Convolutional Neural Networks
Name and reference of original method	• MobileNetV2 • EfficientNetB0 • ResNet50V2 • DenseNet121
Resource availability	Repository name: Betel Leaf Dataset: A Primary Dataset From Field And Controlled Environment Data identification number: 10.17632/btdym2t6mt.1 Direct URL to data: https://data.mendeley.com/datasets/btdym2t6mt/1

## Background

For many developing countries, agriculture plays an important role in the economy, with neat work of farmers and good health of crops at the core of the economy. The trade of betel leaf is one of the highly lucrative businesses, and it also carries considerable economic, cultural, and medicinal importance in the greater part of South Asia, as well as in South-East Asia. It is hindered by damage to the leaves being caused by improper storage, accidental drying, and the spread of disease. The control methods for any disease depend upon the correct leaf specifications. With the advancement in Artificial Intelligence (AI) [1,2], image-based classification for early detection and quality assessment in agriculture-based scenarios has recently surfaced as one of the alternatives over the Deep Learning (DL)-based ones [3]. Recently deep learning has made tremendous strides, which have had a huge impact on the resolution of different data-driven problems across various fields, from medical image analysis using Convolutional Neural Networks [4,5] to phishing website detection in cybersecurity [6]. The cases above support the versatility and strength of AI models in the detection of complicated data patterns, which in turn encourages their use in agricultural image analysis for disease and quality detection.

Many deep learning systems for crop disease classification [7,8] have been deployed, but few have been developed in the case of betel leaves [9]. Most of the previous knowledge that called for study included cereal crops such as rice [[10], [11], [12]], maize, and tomato leaving out economically important crops such as betel. Another issue that stops researchers is the lack of any dataset of betel leaves available in the public domain. There is a lack of datasets containing on-field images, yet model training is conducted using them [13]. Since the event generally typifies post harvest losses and a decrease in market price, proper identification of infected leaves should be done within time or else the same could lead to the transmission of infection on other intact leaves. The purpose of this study is to create a system that can classify betel leaves automatically, accurately, and in a manner that can be comprehended. A formal CNN is taken as a reference to enhance methodological transparency and practical guidance for newcomers in agricultural image analysis.

In this work, the authors propose a deep-learning-based approach to classify the betel leaves into three categories: Healthy, Dried, and Diseased. Also, different from others, owing to the scarcity of any such publicly available datasets, authors prepared their own set of betel-leaf images for this purpose [14,15]. Several deep-learning architectures were then trained and evaluated in search of the best-performing one for the given task. Automatic betel leaf identification is undoubtedly one of the prospective potentials presented as structured data and comparative studies for other works. This study will change the paradigm of research by providing a strong and scalable framework that could help farmers, traders, and researchers in grading and quality assessment of betel leaf and disease management.

## Method details

### Dataset description

The authors used their proprietary data to generate and train the CNN models, which were further deployed for research. This information was made publicly available by Mendeley Data. This dataset was pre-stratified into two operating conditions- Controlled Environment and On-Field. Next, the Dataset enters into the Training and Testing phase, culminating in about a 90–10 % split overall between training and testing, owing to the 10 % validation.

The Controlled Environment domain had an aggregate of 893 images. 803 were channelled into training, while 90 tested the system. Healthy, Diseased, and Dried leaves comprised the training set, which contained 299, 198, and 306 images of each, respectively. The testing set had 34, 22, and 34 images of the same categories.

The On-Field domain had an aggregate of 907 images. 815 were channelled into training, while 92 tested the system. Healthy, Diseased, and Dried leaves comprised the training set, which contained 302, 260, and 253 images of each, respectively. The testing set had 34, 29, and 29 images of the same categories.

**Fig. 1:**
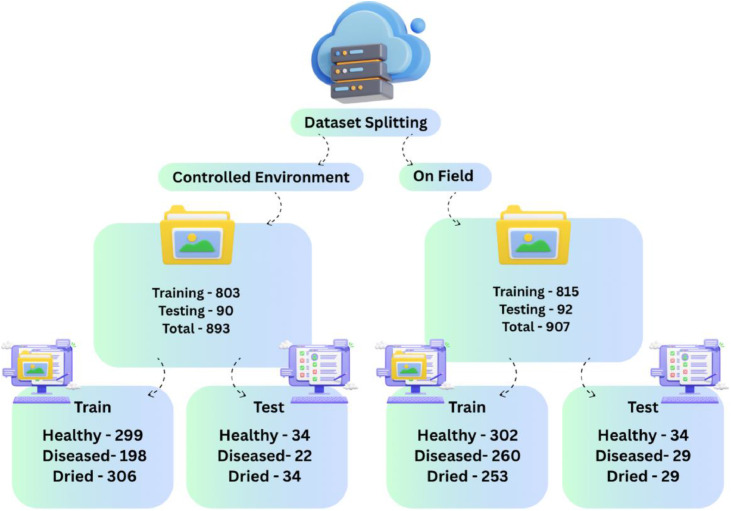
Dataset splitting.

This systematic split gives balanced inclusion of healthy, diseased, and dried leaves in both training and testing subsets as shown in Fig. 1. Therefore, the split forms the appropriate scientific basis for carrying out the correspondence study that will compare the performances of CNN models under Controlled Environment and On-Field scenarios.

**Fig. 2:**
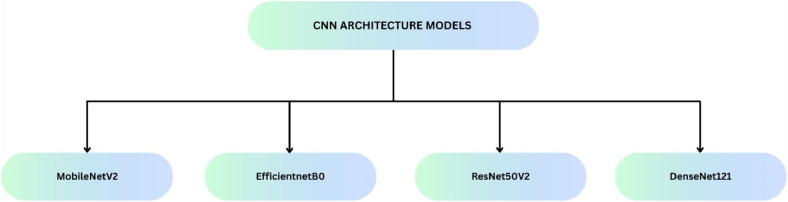
CNN architecture models.

Upon completion of dataset preparation, the dataset was primed for training and evaluation procedures. The authors proceeded with CNN model training on Google Colab through a set of pre-trained architectures, including MobileNetV2, EfficientNetB0, ResNet50V2, and DenseNet121 as shown in Fig. 2. This way, the model was extensively and rigorously tested for leaf images under both Controlled Environment and On-Field conditions.

### CNN pre-trained models

1. MobileNetV2

MobileNetV2 is one of the efficient CNN models made for mobile and embedded vision tasks. One function into a distance pointer convolution to reduce cost by increasing the breadth of cost. The very essence of innovation is an inverted residual block with linear bottlenecks-the shorter connections connecting slimmer layers but not bounding wide ones. Previously, blocks would first compress the input channels before applying a depthwise convolution. The reverse order is now preferred for retaining useful information and attacks representational power: first expand the channels and then apply a depthwise convolution, followed by a final compression via linear layers. MobileNetV2 has about 3.4 million parameters and requires 300 million operations for the standard input size. It achieves very competitive performance, with 72 % Top-1 accuracy on ImageNet classification. It is used in object detection in combination with the SSDLite, which provides less complexity, without much compromise concerning the SSDLite. However, it is also used as a backbone for lightweight semantic segmentation models such as Mobile DeepLabv3. All in all, MobileNetV2 spells a perfect trade-off between accuracy and efficiency, making it a strong candidate for real-time applications on low-resource devices [16].

2. EfficientNetB0

EfficientNetB0 is the convolutional neural network deployed via a neural architecture search for optimizing accuracy and efficiency. It introduces compound scaling in the direction of scaling together depth, width, and resolution, while usually only a single dimension is scaled. An architecture is built using convolution, where different blocks of MBConv each contain a squeeze-and-excite module to cater to the higher extraction of features. The convolution will have a standard convolution first, and then, through stacking of MBConv layers, the spatial size is reduced, and the channel sizes are increased. Then, classification results are produced by a final 1 × 1 convolution, global average pooling, and a fully connected layer. EfficientNetB0 made use of SiLU (Swish-1) activations, which they claim have improved the stability of training. The design allows it to be scaled up systematically to larger models (B1– B7) while still being efficient. Hence, with the given number of parameters and FLOPs, it is much more accurate than traditional CNNs with respect to being a proper candidate for mobile and large-scale problems [17].

3. ResNet50V2

ResNet50V2 is a convolutional neural network built on residual learning, designed to ease the training of very deep models. The novel residual units if combined with identity mappings and pre-activation produce improved residual units, which make the gradient flow during backpropagation smoother. The architecture starts with one convolution and pooling layer and then builds 50 layers in a list connected graph made from a stack of residual blocks. Batch normalization and ReLU are two features applied in each block before the convolutional layer. This makes optimization quite stable. The shortcut identities resist the gradient vanishing. Bottleneck layers (1 × 1, 3 × 3, 1 × 1 convolutions) are the bases for reduction in computation with no loss in accuracy. ResNet50V2 uses preactivation Residual Units, since these have been shown to generalize better and converge faster relative to the original ResNet50. This design enables deep models to scale effectively without performance degradation, making it a powerful backbone for image recognition tasks [18].

4. DenseNet121

DenseNet121-type is a convolutional architectural network interconnecting every layer with one another in a feed forward manner. Normally, architectures pass feature maps of only immediately preceding layers to subsequent layers, whereas DenseNet121 keeps feature maps of all preceding layers and passes them on to all subsequent layers. In this way, feature reuse is encouraged, and a smooth gradient flow can also be maintained. The model is composed of dense blocks, each of which comprises a number of convolutional layers. In these blocks, the outputs are concatenated and not summed. Transition layers, together with batch normalization, 1 × 1 convolutions, and pooling operations, are located between dense blocks in order to control the dimension. With such a design, redundancy in parameters is reduced to the minimum, the total number of parameters is kept low, but the lowering of the vanishing gradient is somewhat apparent. DenseNet121 uses four dense blocks followed by a global average pooling layer and a fully connected classification layer. Besides, the network utilizes the bottleneck layers and compression to enhance efficiency. The compact geometry and deep layers deliver very high accuracy with fewer parameters than usual CNNs. DenseNet121 is often deployed in tasks where image recognition is important because of its satisfactory working capacity and cost being very minimal [19].

### Common setup

Across all four models, the training dataset was prepared by splitting the data, from which 90 % of the split was given for training (and a 10 % validation set was carved out) while the remaining 10 % was held for testing with ImageDataGenerator of Keras, and every image's pixels were divided by 1/255 for normalizing. The images were resized to 224 × 224 pixels with 3 channels (RGB) each as shown in Table 1, as per the requirements of all models. While training, the batch size was kept as 32 for a proper balance between computation cost and convergence of the model, and epochs were kept at 32 for accuracy monitoring on the validation split. Adam was used as the optimizer for network compiling with categorical cross-entropy as the loss objective, and accuracy was the main metric used for evaluation and comparison [20]. The models were initialized with ImageNet pretrained weights [21], removing the top classification layer and adding a GAP (Global Average Pooling) layer followed by a Dense layer with softmax activation for classification into 3 classes: Healthy, Dried, or Diseased. A detailed comparison of the transfer learning model configurations is provided in Table 2.

**Table 1:** Comparison of original and resized images (224 × 224).

**Table 2:** Comparison of transfer learning model configurations.

Configuration Detail	MobileNetV2	EfficientNetB0	ResNet50V2	DenseNet121
Input Size	224 × 224 × 3 (RGB)	224 × 224 × 3 (RGB)	224 × 224 × 3 (RGB)	224 × 224 × 3 (RGB)
Pretrained Weights	ImageNet	ImageNet	ImageNet	ImageNet
Conv Base Trainable	Frozen	Fully Trainable	Frozen	Fully Trainable
Added Layers	GAP → Dense(3)	GAP → Dense(128, ReLU) → Dense(3)	GAP → Dense(128, ReLU) → Dense(3)	GAP → Dense(128, ReLU) → Dense(3)
Activation(s)	Softmax	ReLU, Softmax	ReLU, Softmax	ReLU, Softmax
Final Output Layer	Dense(3, Softmax)	Dense(3, Softmax)	Dense(3, Softmax)	Dense(3, Softmax)
Optimizer & Loss	Adam & Categorical Cross-Entropy	Adam & Categorical Cross-Entropy	Adam & Categorical Cross-Entropy	Adam & Categorical Cross-Entropy
Batch Size	32	32	32	32
Epochs	32	32	32	32
Classes	3 (Healthy, Dried, Diseased)	3 (Healthy, Dried, Diseased)	3 (Healthy, Dried, Diseased)	3 (Healthy, Dried, Diseased)

### Model-Specific configurations

1. MobileNetV2 was configured with frozen convolutional base layers to prevent overfitting, allowing only the newly added classifier layers to be trained as shown in Fig. 3.

**Fig. 3:**
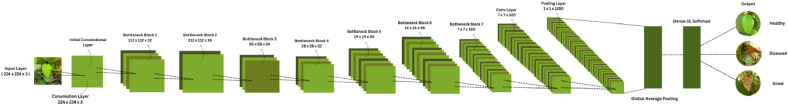
MobileNet architecture.
2. EfficientNetB0 included an additional dense layer of 128 units with ReLU activation before the final softmax layer, and its convolutional base was kept fully trainable to enable fine-tuning of the entire network as shown in Fig. 4.

**Fig. 4:**
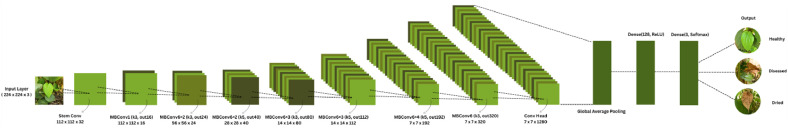
EfficientNetB0 architecture.
3. ResNet50V2 also incorporated a 128-unit dense layer with ReLU, but its convolutional base layers were frozen, making it function as a fixed feature extractor as shown in Fig. 5.

**Fig. 5:**
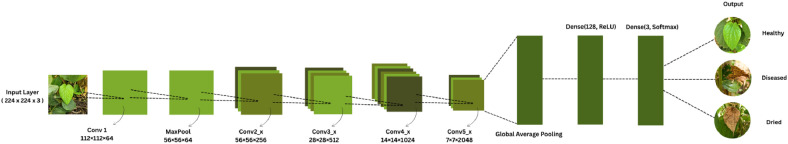
ResNet50V2 architecture.
4. DenseNet121 followed the same architecture modification as EfficientNetB0 (a 128-unit dense layer with ReLU before the classifier) and was fully fine-tuned by keeping its convolutional layers trainable as shown in Fig. 6.

**Fig. 6:**
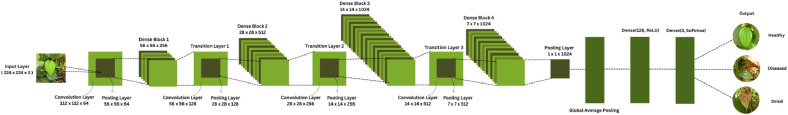
DenseNet121 architecture.

An ablation study was performed to analyze the impact of image acquisition conditions on computational performance by individually training each backbone model using images taken under Controlled Environment (CE) and On-Field (OF) conditions. All the models shared the same training configuration and hardware setup and were trained for 32 epochs. The results illustrate the effect of changed image characteristics during real-world conditions in OF on the training time and inference time as compared to CE images while model architecture remained the same as shown in Table 3. To provide a fair comparison, all models were trained and evaluated on Google Colab (Runtime Type – Python 3, Hardware accelerator – CPU) in the same hardware environment. Training time was taken as the total wall-clock time required to go through 32 epochs of our dataset, and inference time as the total time required to perform forward passes through the entire test set with a fixed batch size. Training time is stated in minutes because of its large extent, while inference time is given in seconds to indicate deployment-level latency. The reported times are an excellent means of comparative evaluation regarding the computational efficiency of the models evaluated because all experiments were performed under similar conditions. In addition, the trainable, non-trainable, and total parameter statistics of each architecture are summarized in Table 4.

**Table 3:** Computational complexity analysis.

Model	Type	Training Epochs	Training Time (In Minutes)	Inference Time (In Seconds)
MobileNetV2	CE	32	133.75	30
	OF		154.58	41
EfficientNetB0	CE	32	119.16	23
	OF		148.03	24
ResNet50V2	CE	32	97.18	30
	OF		111.1	26
DenseNet121	CE	32	421.73	48
	OF		401.58	52

**Table 4:** Model parameter statistics of the evaluated CNN architectures.

Model	Trainable Parameters	Non-Trainable Parameters	Total Parameters
MobileNetV2	3843	2257,984	2261,827
EfficientNetB0	4171,903	42,023	4213,926
ResNet50V2	262,659	23,564,800	23,827,459
DenseNet121	7085,443	83,648	7169,091

To analyze the similarity and dissimilarity of learned representations across different architectures, the authors decided to open the outputs of mid-level layers in each network that corresponded to one another without enforcing any dimensional alignment. The layers authors selected create feature maps with the same spatial resolution (14 × 14), which means that there is an equal coverage of receptive field and spatial representation across models. The only difference is that the number of channels is different; MobileNetV2 Bottleneck Block 5 gives out 14 × 14 × 64, EfficientNetB0 MBConv6 × 3 (k5) gives out 14 × 14 × 112, ResNet50V2 Conv4_x gives out 14 × 14 × 1024, and DenseNet121 Dense Block 3 gives out 14 × 14 × 1024. The variations in channel dimensionality are due to the variations in architectural designs, and these differences were deliberately kept in order to study the different models capabilities of mid-level features encoding at the same spatial resolution.

## Method validation

### Results

After training, the performance of the CNN models: MobileNetV2, EfficientNetB0, ResNet50V2, and DenseNet121 was neatly evaluated on the dataset with metrics such as Accuracy, Precision, Recall, and F1-score. As shown in Table 5, the evaluation values are mentioned. The DenseNet121 model had the best values among the other metrics, not only on the Controlled Environment set but also, more importantly, on the On-Field set. From this comparison, one can learn that the model EfficientNetB0 shows poor performance; its accuracy, precision, recall, and F1-score are relatively lower in both sets. On the contrary, DenseNet121 appears to be the best model throughout, excelling with high performance at distinguishing between healthy, diseased, and dried leaves. Therefore, DenseNet121 emerges as the most reliable architecture for this classification task; furthermore, this also opens up avenues for reworking or strengthening EfficientNetB0, which can be either by hyperparameter tuning or changing the architecture or training with a larger and more diverse dataset, so that it performs equally well.

**Table 5:** Comparison of CNN models for evaluation.

Model	Type	Accuracy	Precision	Recall	F1 Score
MobileNetV2	CE OF	97.777778	97.962963	97.777778	97.792417
		90.217391	90.116098	90.217391	90.098344
EfficientNetB0	CE OF	37.777778	14.271605	37.777778	20.716846
		36.956522	13.657845	36.956522	19.944790
ResNet50V2	CE OF	97.777778	97.901235	97.777778	97.756614
		84.782609	85.031946	84.782609	84.844720
DenseNet121	CE OF	92.222222	92.431389	92.222222	92.212183
		96.739130	96.896135	96.739130	96.690465

To study the model behaviour neatly with respect to class level predictions, confusion matrices are added below in Table 6 for each model in the two categories, Controlled Environment and On-Field. Each metric shows each test image was predicted into which category and was of which actual category. With this comparison of confusion matrices, anyone can assess their overall prediction strength, class wise robustness, and potential biases.

**Table 6:** Classification confusion matrix results.

**Fig. 7:**
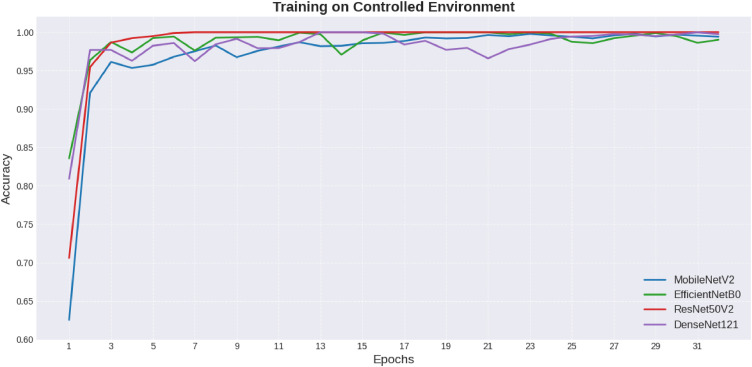
Comparison of models on controlled environment.

**Fig. 8:**
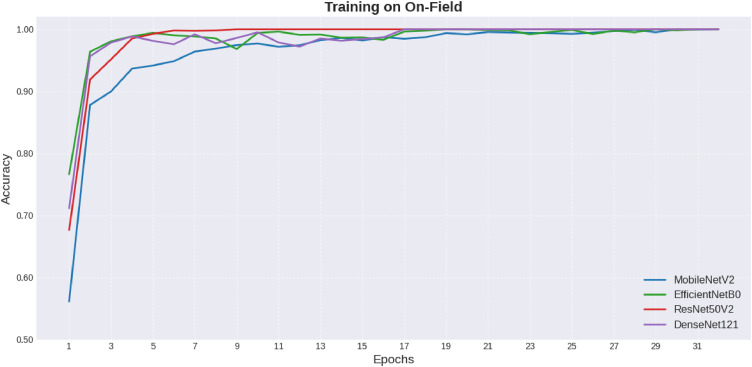
Comparison of models on-field.

Continuing, the development of training and validation accuracies through the epochs in the case of the Controlled Environment and On-Field datasets is shown in Fig. 7, Fig. 8, respectively. Training charts show the trend for training stability, where DenseNet121 reveals the model that produced the best results consistently.

### Limitations

While all four CNN architectures were able to deliver high performance (90–97 % across accuracy, precision, recall, and F1-score), EfficientNetB0, however, was the one that stood out with the least results. This is likely due to the fact that its convolutional base is fully trainable, which in turn may have led to overfitting of the limited betel leaf dataset. Additionally, its compound scaling method makes it extremely dependent on the learning rate and fine-tuning xparameters. The uniform training setup for all models (epochs, optimizer, batch size) might not have been the best for each individual architecture. Besides, the limited size of the dataset might have influenced model generalization as well as class sensitivity. Due to computational constraints, deeper experiments such as hyperparameter tuning and cross-validation were not possible. Moreover, the absence of interpretability in the models restricts the understanding of feature learning.

Next, researchers may consider progressive fine-tuning and differential learning rates for EfficientNetB0. By adding data augmentation and using larger and more diverse datasets, the model's robustness could be improved. Furthermore, using explainable AI techniques can give more transparency and trust to model decisions. On top of that, testing models under real-world agricultural environments will not only validate practical reliability but also trustworthiness. Also, cross-domain evaluation on other plant species can help generalization to be extended. Finally, combining CNNs with attention mechanisms may be a viable way to achieve even higher accuracies. These future works not only can enhance model capabilities but also the application potential in the field of agricultural AI.

## Related research article

Mane, G., Bhise, R., Kadam, R., Kaur, G., Verma, D., Chopade, R., Shinde, G., Tejani, G. G., & Mousavirad, S. J. (2025). PriBeL: A primary betel leaf dataset from field and controlled environment. *Data in Brief, 61*. https://doi.org/10.1016/j.dib.2025.111674

## For a published article

Mane, G., Bhise, R., Kadam, R., Kaur, G., Verma, D., Chopade, R., Shinde, G., Tejani, G. G., & Mousavirad, S. J. (2025). PriBeL: A primary betel leaf dataset from field and controlled environment. *Data in Brief, 61*. https://doi.org/10.1016/j.dib.2025.111674

## CRediT author statement

Gauri Mane: Conceptualization, Writing – Original Draft, Writing – review & editing, Methodology, Software;

Raghav Bhise: Conceptualization, Writing – Original Draft, Writing – review & editing, Methodology, Software;

Rutuja Kadam: Supervision, Validation; Gagandeep Kaur: Reviewing, Editing; Gitanjali Shinde: Supervision, Validation; Grishma Bobhate: Reviewing; Sonal Fatangare: Supervision, Validation.

## Ethics statements

None.

## Declaration of competing interest

The authors declare that they have no known competing financial interests or personal relationships that could have appeared to influence the work reported in this paper.

## Data Availability

I have shared link to my code at the Attach File step.

## Appendix Supplementary materials

Image, application 1
