# Supplementary material for: PriBeL-Net: Extending betel leaf dataset with CNN-based image classification
Source: MethodsX. 2026 Feb 15;16:103828. doi: 10.1016/j.mex.2026.103828 (PMC12950366; doi:10.1016/j.mex.2026.103828)
Supplement: Supplementary file 1 [file mmc1.docx]

<https://github.com/raghavbhise/Model-Training-and-Evaluation>
